# Successful palliative approach with high-intensity focused ultrasound in a patient with metastatic anaplastic pancreatic carcinoma: a case report

**DOI:** 10.3332/ecancer.2016.635

**Published:** 2016-04-21

**Authors:** Antonio Ungaro, Franco Orsi, Chiara Casadio, Salvatore Galdy, Francesca Spada, Chiara Alessandra Cella, Clementina Di Tonno, Guido Bonomo, Paolo Della Vigna, Sabina Murgioni, Anna Maria Frezza, Nicola Fazio

**Affiliations:** 1 Unit of Gastrointestinal Medical Oncology and Neuroendocrine Tumours, European Institute of Oncology, Milan 20141, Italy; 2 Unit of Diagnostic Cytology, European Institute of Oncology, Milan 20141, Italy; 3 Unit of Interventional Radiology, European Institute of Oncology, Milan 20141, Italy

**Keywords:** anaplastic pancreatic carcinoma, HIFU, palliative treatment, abscopal effect

## Abstract

We report a case of a 74-year-old man with a metastatic anaplastic pancreatic carcinoma (APC). After an early tumour progression on first-line chemotherapy with cisplatin and gemcitabine, even though it was badly tolerated, he was treated with a combination of systemic modified FOLFIRI and high-intensity focused ultrasound (HIFU) on the pancreatic mass. A tumour showing partial response with a clinical benefit was obtained. HIFU was preferred to radiotherapy because of its shorter course and minimal side effects, in order to improve the patient’s clinical conditions.

The patient is currently on chemotherapy, asymptomatic with a good performance status.

In referral centres, with specific expertise, HIFU could be safely and successfully combined with systemic chemotherapy for treatment of metastatic pancreatic carcinoma.

## Introduction

Anaplastic pancreatic carcinoma (APC) is a highly malignant cancer arising from epithelial lineage. It has been reported to have a high capacity to infiltrate tissues and a strong tendency to metastasise [1]. Prognosis is poor with a five-month median survival time since diagnosis and <1% five-year survival rate, irrespective of anti-tumour treatment [2]. Resective surgery if feasible, systemic chemotherapy, and chemo-radiation are usually proposed, unfortunately with a low impact on global prognosis [3].

## Case report

A 74-year-old man with a history of dyslipidaemia, impaired glucose tolerance, and cholelithiasis, came to our attention because of dyspepsia and dorsal pain lasting for two months. He underwent an upper gastrointestinal (GI) endoscopy showing a submucosal nodule in the gastric antrum, and an abdomen ultrasonography (US) showing multiple gallstones and a 3 cm pancreatic body lesion.

A chest-abdomen contrast medium Computed Tomography (CT)-scan detected a pancreatic body lesion of 26 mm axial diameter (Ø) with pancreatic duct dilation and perilesional lymphadenopathy with 15 mm Ø. Endoscopic US (EUS) with fine-needle aspiration (FNA) showed a 37 mm Ø hypoechoic and inhomogeneous lesion comprising mesenteric-portal axis without a clear infiltration (uT3uN0, AJCC 2010) [4]. Cytology was conclusive for anaplastic/undifferentiated pancreatic carcinoma. Immunohistochemical (IHC) analysis was positive for cytokeratin AE1-AE2, negative for S-100 protein, Melan-A, chromogranin-A (CgA), synaptophysin (Syn), and CD-56.

**Figure 1.**
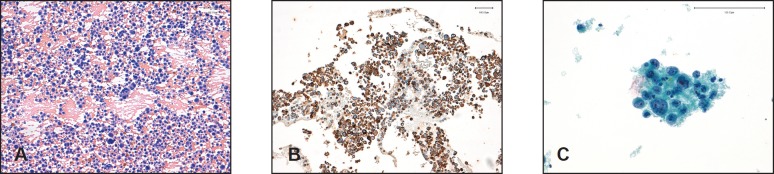
Haematoxilin/eosin-stained pancreatic cytology revealed the presence (A) of poorly cohesive, pleomorphic, monucleated or multinucleated large cells (20x). Positivity (B) for cytokeratins AE–AE2 confirms the diagnosis of anaplastic cell carcinoma (20x). Peritoneal washing cytology (PWC) with Papanicolaou stain (C) detects cells with malignant features such as nuclear displacement, irregular nuclear membranes, small and eccentric nucleoli (40x).

**Figure 2.**
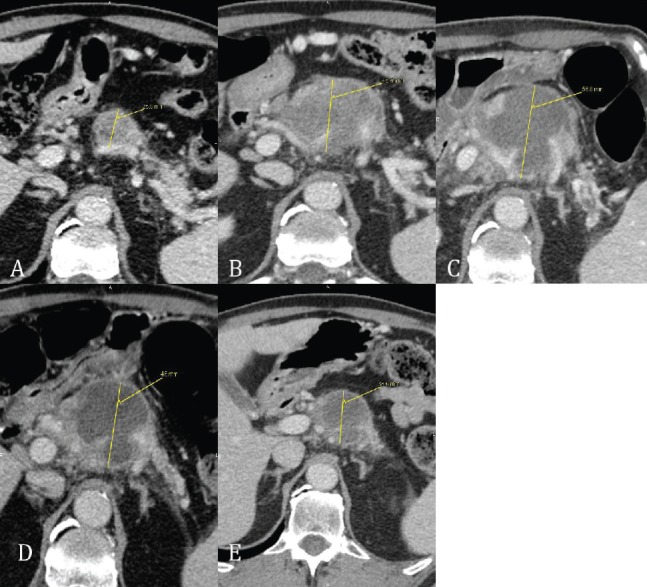
(A) Baseline CT scan revealed an inhomogeneous hypodense lesion in the head of pancreas; (B) after chemotherapy with gemcitabine plus cisplatin; (C) within 24 hours after HIFU pancreatic lesion shows a lump of necrotic tissue (between thrid and fourth FOLFIRI cycle); (D) CT scan at six weeks after HIFU; (E) 16 weeks after HIFU.

On this basis after discussion within the gastrointestinal multidisciplinary team (GI-MDT), a diagnostic laparoscopy plus intraoperative US were performed showing no evidence of peritoneal carcinomatosis but infiltration of splenic artery and upper mesenteric vein were seen. A peritoneal washing cytology (PWC) revealed the presence of neoplastic cells (Figure 1). Following a further multidisciplinary discussion, systemic chemotherapy was therefore proposed. The patient was asymptomatic; his performance status (PS) was 0 Eastern Cooperative Oncology Group (ECOG). Because of the particular histotype, he was not eligible for first-line chemotherapy trials, therefore a regimen with cisplatin (60 mg/mq day 1 every 21) and gemcitabine (1000 mg/mq days 1, 8, every 21) was decided on an individual scale and administered outside clinical trials (Figure 2). After day one of the second cycle, treatment was delayed because of thrombocytopaenia, and definitively discontinued because of unstable clinical status for acute lower limb peripheral neuropathy and appearance of upper abdominal pain. In the meantime, given the time from the start of chemotherapy, a restaging C-scan was performed, showing an increased pancreatic lesion (46 mm Ø) with dilatation of the Wirsung duct, and multiple pathological lymph nodes in the retroperitoneal region. As a result, we assumed that the patient had a metastatic disease, based on the radiological features (morphology, dimension, rapid growth, and contrast enhancement of paraaortic lymph nodes) and clinical evaluation (increased level of Ca 19.9, deterioration of the patient’s clinical condition and the previously positive peritoneal cytology). On this basis a third GI-MDT discussion indicated a second-line systemic chemotherapy with modified FOLFIRI (folinic acid 200 mg/mq; irinotecan 180 mg/mq; bolus fluorouracil (5-FU) 400 mg/mq; continuous infusion 5-FU 2400 mg/mq) at 70% of the conventional dose.

Furthermore, because of the particular pattern of progression (expansive rather than infiltrating primary tumour growth) and its local symptomaticity, a locoregional palliative procedure was discussed.

Therefore, after the fourth cycle of chemotherapy the patient underwent high-intensity focused ultrasound (HIFU) ablation on the pancreatic lesion. The treatment was performed with computerised axial scanning and power Doppler to evaluate the effect of ablation on the artery engaged. The patient was placed in a prone position on the HIFU table, and then we verified that the abdomen skin was in very close contact with degassed water. The treatment was performed with vertical scanning mode separated by 5mm-distance between each slice. HIFU ablation was performed using the JC-HIFU system (Chongquing Haifu-HIFU-Tech, Chongquing, China). The procedure was supervised by real time US; the diameter of transducer that issues US energy was 20 cm with a focal length of 15 cm and a frequency of 0.8 MHz. The software used for the real time imaging unit was MyLab70 imaging device (Esaote, Genova, Italy). This 1.0 to 8.0 MHz imaging probe is located in the centre of the HIFU transducer. Treatment was carefully monitored with US guide and finished when the lesion showed a large part of coagulation necrosis in the centre. The duration of HIFU ablation was about 90’. At the end, US revealed an extended necrosis area on the pancreatic lesion. A CT-scan performed the day after revealed a 56 mm Ø pancreatic lesion and paraaortic lymphadenopathies. After HIFU the patient received five more cycles of modified FOLFIRI. A CT-scan performed six weeks after HIFU revealed a reduction of the pancreatic head lesion (45 mm versus 56 mm Ø) and of the left paraaortic lymph nodes (15 mm versus 33 mm Ø). A restaging CT-scan after four months from the HIFU treatment revealed a further reduction of the pancreatic lesion (38 mm versus 45 mm Ø) and left paraaortic lymph nodes (8.4 mm versus 15 mm Ø).

The patient became totally asymptomatic after HIFU. Chemotherapy was well tolerated and is still ongoing.

## Discussion

Clinical presentation of APC is generally unspecific, including abdominal pain, vomiting, anorexia, and weight loss [5]. Furthermore, APC can cause local complications such as bleeding and obstructive jaundice.

In the absence of literature data, a first-line therapy with gemcitabine plus cisplatin was delivered without benefit as shown from clinical worsening and radiological disease progression [6]. Furthermore its feasibility was hard because of several delays and toxicity.

Therefore, a combination of fluoropyrimidine and irinotecan was proposed, based on the literature data derived from the activity of camptothecin-based therapy after platinum-based therapy in pancreatic adenocarcinoma and also on the different toxicity profile of this regimen compared with the previous one [7]. Because of the particular pattern of progression, which was mainly locoregional, with expansive rather than infiltrating picture and it being locally symptomatic, a locoregional treatment was thought would add something in terms of both local tumour growth control and quality of life (QoL) improvement.

The most common palliative locoregional treatment used in pancreatic cancer is external radiotherapy (RT). Sporadic literature data exist on intra-arterial chemotherapy [8]. Use of RT in metastatic disease is required for pain control with a palliative dose of 30 Gy. This treatment can also delay local tumour progression, but reduction is not expected. On the other hand, potential gastrointestinal toxicity induced by radiotherapy, duodenum perforation, and haematologic disorders are possible when large tumour masses are treated [9].

We chose HIFU in order to control both cancer related symptoms and reduction of local disease. Because of a shorter duration compared with radiotherapy, a less invasive approach compared to intra-arterial chemotherapy and our local interventional radiology expertise [10, 11, 12], we believed that HIFU would be the most feasible, potentially effective, and a safer approach.

HIFU ablation is a non-invasive technique used for the control of localised tumour mass. It uses focused ultrasound energy to induce high temperature in the tumour and then induce necrosis and apoptosis of malignant cells. Acoustic energy is absorbed by focused target and high temperature is generated within it, but not outside the region. It is not necessary to perform any incision but light sedation or anesthesia is often suggested [13, 14].

HIFU efficacy on tumour growth control is still largely debated. In a Chinese study including 251 patients treated with HIFU for advanced pancreatic carcinoma, the majority of those with locally advanced unresectable stage had an improvement of abdominal pain control and local symptoms because of reduction in tumour mass size [15].

HIFU treatment differs from other locoregional treatments, such as radiotherapy or neurolysis procedure, because it requires a single session rather than multiple ones and it is not invasive.

In pancreatic cancer patients pain control is one of the most important clinical issues, usually achieved by means of opioids. A minority of patients are resistant and local treatments are usually considered. Generally more often alcohol neurolysis or external radiotherapy are considered [16]. After a multidisciplinary discussion involving interventional radiologists, surgeons, and medical oncologists, we decided to choose HIFU for the aforementioned reasons because of its one-shot technique, its previous favourable results in unresectable pancreatic cancer, and its greater suitability to be combined with chemotherapy when compared with radiation therapy. Indeed HIFU did not condition the global plan of FOLFIRI chemotherapy.

This case suggests that a multidisciplinary discussion can be useful and beneficial for patients even in a context of metastatic poor prognosis malignancy, like APC.

Palliative care is a mainstay of the treatment for patients with advanced pancreatic cancer. Other than improving global QoL, it can also improve the tolerability of chemotherapy, the compliance of the patient to antitumour therapy thereby making systemic chemotherapy more feasible. In general it could even improve overall survival (OS). Improving palliative care in this clinical context means increasing the possibility that patients receiving more lines of systemic chemotherapy and possibly they could obtain an improvement of both QoL and mood, although this has not yet been demonstrated in pancreatic cancer [17].

On the other hand, HIFU ablation in this patient has had a significant effect in the control of local disease and more effect has been described in distant sites too, (paraaortic lymph nodes) away from the treated area.

This therapeutic benefit, also called ‘abscopal effect’ (from the Latin ‘ab’-position away from-and ‘scopus’-target-), has been observed and described mostly in radiation therapy [18, 19], but not in other local treatments such as HIFU ablation.

In line with the definition of abscopal effect, tumour regression is usually observed several months after treatment as a result of immunomediated events. Probably this type of cancer cell death occurs from immunogenic processes, according to the recent discovery underlying the importance of immunomodulated toxicity, especially observed with the introduction in clinical pratice of checkpoint inhibitors (combined with radiation therapy or not). Nevertheless, HIFU treatment has to be assessed in clinical studies to evaluate the potential immunomodulating effect combined or not with systemic therapy or genetic modifications which can promote this local treatment.

## Conclusion

In conclusion, in advanced pancreatic carcinoma, HIFU can have a role in locally advanced unresectable disease or even in a metastatic setting, both for tumour growth control and to improve symptom control and therefore QoL. Furthermore, it can be easily combined with systemic chemotherapy. According to our case report, HIFU should be included in the multidisciplinary discussion of symptomatic patients with metastatic pancreatic cancer, i.e. is to be combined with systemic treatment. This of course will require skilled operators and therefore it should be considered only in referral institutions which have GI-MDT and interventional radiology with high expertise.
